# Adaptive Inertial Sensor-Based Step Length Estimation Model

**DOI:** 10.3390/s22239452

**Published:** 2022-12-03

**Authors:** Melanija Vezočnik, Matjaz B. Juric

**Affiliations:** Faculty of Computer and Information Science, University of Ljubljana, Večna Pot 113, 1000 Ljubljana, Slovenia

**Keywords:** accelerometer, inertial sensing, smartphone, step length estimation model

## Abstract

Pedestrian dead reckoning (PDR) using inertial sensors has paved the way for developing several approaches to step length estimation. In particular, emerging step length estimation models are readily available to be utilized on smartphones, yet they are seldom formulated considering the kinematics of the human body during walking in combination with measured step lengths. We present a new step length estimation model based on the acceleration magnitude and step frequency inputs herein. Spatial positions of anatomical landmarks on the human body during walking, tracked by an optical measurement system, were utilized in the derivation process. We evaluated the performance of the proposed model using our publicly available dataset that includes measurements collected for two types of walking modes, i.e., walking on a treadmill and rectangular-shaped test polygon. The proposed model achieved an overall mean absolute error (MAE) of 5.64 cm on the treadmill and an overall mean walked distance error of 4.55% on the test polygon, outperforming all the models selected for the comparison. The proposed model was also least affected by walking speed and is unaffected by smartphone orientation. Due to its promising results and favorable characteristics, it could present an appealing alternative for step length estimation in PDR-based approaches.

## 1. Introduction

The continuous advances in the field of microelectromechanical technology have led to the mass production of sensors. Inertial sensors are becoming ubiquitous as many applications are readily available [1]. Their presence is not only limited to Internet-of-Things (IoT) devices, such as smartphones, tablets, or smartwatches, but it is also emerging in the fields of automotive and aerospace industries as it enables more accurate position estimation [2]. Nevertheless, researching applications of IoT inertial sensors presents a very appealing means, as global smartphone market sales reached 1.4 billion worldwide last year [3]. Researchers have already studied a plethora of different inertial sensor applications, including health applications such as the gait of patients who suffered a stroke [4,5,6,7], Parkinson’s disease [8,9,10,11,12,13,14], or Huntington’s disease [15,16,17]. Another set of studies is related to monitoring the person’s activity by means of gait authentication [18,19,20,21,22,23], activity recognition [24,25,26,27,28,29], and PDR [30,31,32,33,34,35,36].

Approaches to PDR determine the current position by estimating the change in the previous position, usually based on step length and heading. In particular, a range of different techniques is known to be employed for the step length estimation process. Various machine learning techniques and statistical estimation methods are often used to achieve higher accuracy, yet they depend upon comprehensive training. For instance, artificial neural networks [37] and Bayesian filters, such as particle filters [38,39,40], grid-based approaches [41], and Kalman filters [42,43], have already been employed. Integrating acceleration in the walking direction with respect to time [11,44,45] presents another means of estimating step length. As inertial sensors are prone to errors caused by interference, noise, and instability, drift has to be eliminated during the estimation process [46,47]. Moreover, these techniques often require sensor placement on the center of body mass, shank, or foot. Utilizing a step length estimation model presents another means of determining step length [32,33,34]. This procedure is frequently utilized for estimating step length on smartphones when considering PDR [48]. For this reason, we aimed our work at advancing the field of step length estimation models. 

Step length estimation models usually calculate step length based on a linear combination of inertial sensor measurements, such as step frequency or acceleration. Some models exploit the relationship between step length and step frequency [34,49,50,51]. For example, the model proposed by Alvarez et al. [49] employs the linear relationship. In addition, Renaudin et al. [50] extended this linear relationship with the user’s height, while Zhang et al. [51] included the user’s leg length. Tian et al. [34] exploited the relationship between step length, the square root of step frequency, and user’s height. A number of step length estimation models consider a physical model of gait, such as the inverted pendulum model [52], as the basis. These models often exploit the relationship between acceleration and step length. Zijlstra and Hof [53], Lan and Shih [54], and Do et al. [32] based their models on the vertical displacement of the center of body mass and included the user’s leg length in the models. In contrast, Weinberg [55] used the difference between maximum and minimum acceleration values within a step as the basis, whereas Kim et al. [56] utilized the mean absolute acceleration value in the walking direction within the step. Vezočnik et al. [57] based their model on the difference between maximum and minimum acceleration magnitude values within the step. Guo et al. [58] and Bylemans et al. [31] extended the model proposed by Kim et al. [56]. Guo et al. [58] included another tunable constant, whereas Bylemans et al. [31] included step frequency and the difference between the maximum and minimum vertical acceleration values within the step. Similarly, Mikov et al. [59] and Kang and Han [33] extended the Weinberg model [55]. Mikov et al. [59] included the inverse of step frequency while Kang and Han [33] included another tunable constant and also employed logarithm for step length estimation. Shin and Park [60] utilized step frequency and acceleration magnitude variance in the model. Sharp and Yu [61] based their model on step frequency, the user’s height, and the difference between the maximum and minimum vertical acceleration values within the step. 

In one of our previous studies, we conducted an in-depth analysis and comparison of representative step length estimation models and demonstrated that the performances of certain models change under different evaluation protocols when smartphone placements and walking speeds vary in trials [48]. Moreover, authors also rarely consider the kinematics of different body segments during walking and step lengths in the derivation process of the model [57]. Specifically, identifying similar characteristics related to the movement of anatomical landmarks by means of the kinematics of the human body during walking does not often lay the groundwork for a new model, as it did in our previous study in which we proposed a step length estimation model based on the difference between maximum and minimum acceleration magnitude values within the step [57]. In addition, we aimed to include the minimum number of input parameters in the model and compare the evaluation results of the proposed model against models that exhibit similar composition, i.e., step length is calculated as the product of a tunable constant and one input parameter. The proposed model produced an overall MAE of 6.44 cm for treadmill walking, outperforming all the models selected for the comparison. Nevertheless, the proposed model achieved less promising results for free walking when compared to the step-frequency-based model, but it was the least affected by walking speed and smartphone position among the acceleration-based models selected for the comparison in that study.

For these reasons, we aimed to investigate the possibilities of extending the acceleration-based model we proposed in our previous work when considering the movement of anatomical landmarks during walking [57]. As the base of the model already includes acceleration magnitude input, we utilized canonical correlation analysis (CCA) to investigate the link between acceleration magnitude, step length, and other characteristics defining the movement of anatomical landmarks, resulting in the adaptive step length estimation model herein based on step frequency and acceleration magnitude inputs. This model is, therefore, unaffected by smartphone orientation, unlike several acceleration-based models. Another aim of this work was to evaluate the proposed model for different walking speeds and smartphone positions. As a consequence, we utilized the publicly available dataset that includes inertial sensor measurements acquired by four off-the-shelf smartphones for both treadmill walking and free walking [62]. The optical measurement system tracked the spatial positions of the smartphones during treadmill experiments. Moreover, it also aided in determining the walked distance during free walking.

## 2. Materials and Methods

Proposing a new step length estimation model typically entails a two-stage process consisting of a derivation and evaluation of the model. The scope of the former stage often combines inertial sensor data acquisition and analysis of these data. Obtained insights from this analysis lay the basis for a new model, which is evaluated next. Evaluation datasets are usually larger and more diverse between trials in terms of sensor position, terrain, walking speed, and duration. 

In our previous work, we derived a new model by identifying similar characteristics of the movement of anatomical landmarks by means of the kinematics of the human body during walking [57]. The proposed model utilizes acceleration magnitude as the main input and is, consequently, unaffected by smartphone orientation, yet its performance was affected by walking speed. This study aimed to eliminate previously identified limitations of the model by investigating the possibilities of including additional input parameters. We utilized CCA in this process to support the choice of the parameters before proposing a new model that we evaluated at last. 

### 2.1. Design of the Study

**Figure 1:**
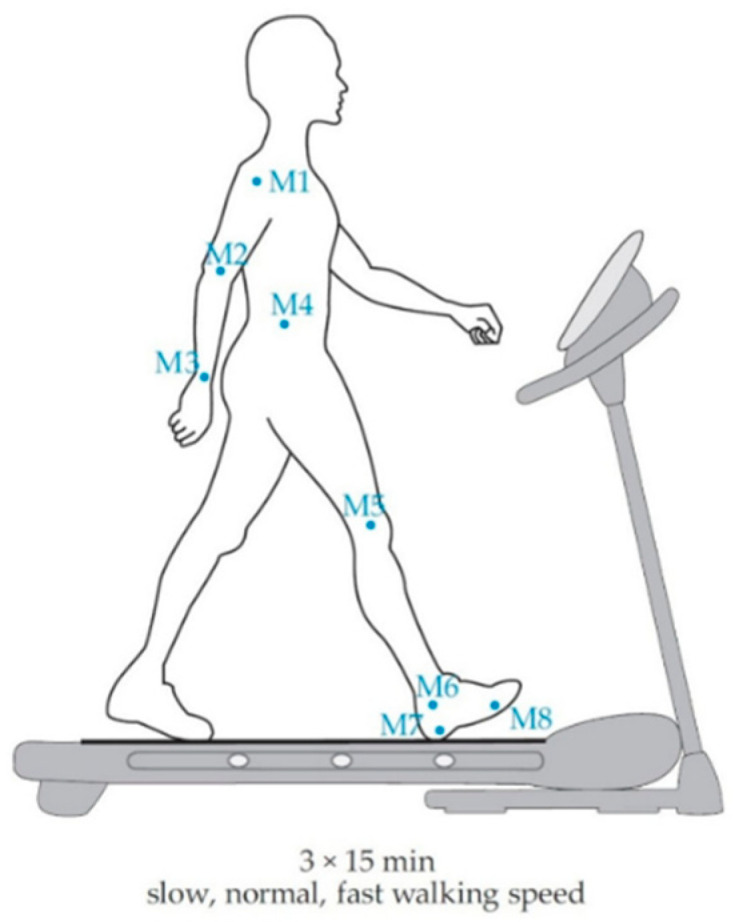
Derivation of the model [57].

**Figure 2:**
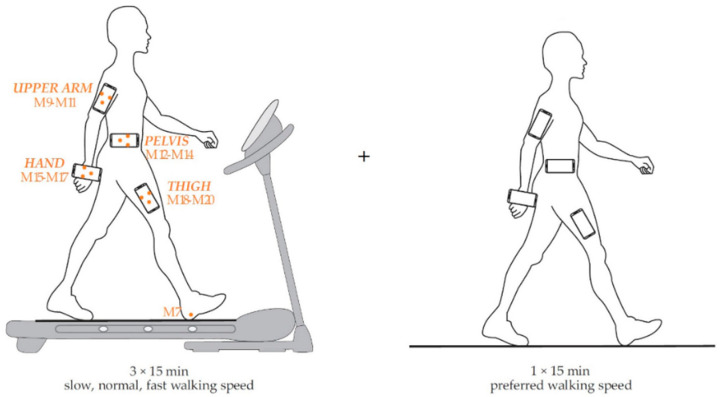
Performance evaluation of the model [57].

The aim of this work was to eliminate previously identified limitations of the model [57], i.e., reducing the impact of the walking speed on the model, and to evaluate the new model. The first stage of this study was, therefore, dedicated to investigating the potential extensions of our previous model while considering the movement of anatomical landmarks during long-term walking, as one of our aims was to propose a model suitable for different smartphone placements. For this reason, we adopted a similar design of the study for the derivation and evaluation of the model as shown in Figure 1 and Figure 2, respectively [57]. 

Experimental protocol and data analysis are presented in detail in our previous work [57], and only essential aspects are highlighted herein. The walking of healthy adults can be considered symmetrical regardless the gender [63]. As a consequence, the motion on one side of the human body was investigated by considering gait cycles and stride lengths of one limb. Anatomical landmark positions were measured by infrared markers using the optical motion capture system Optotrak Certus (Northern Digital Inc., Waterloo, ON, Canada). Infrared markers Mi, 1 ≤ i ≤ 8, were attached to the anatomical landmarks (as shown in Figure 1) as follows. The infrared marker M1 was attached to the shoulder (acromion process), the infrared marker M2 was attached to the elbow (lateral epicondyles), the infrared marker M3 was attached to the wrist (ulnar styloid process), the infrared marker M4 was attached between the hip (greater trochanter) and the pelvis (upper iliac crest), the infrared marker M5 was attached to the knee (lateral femoral condyle), the infrared marker M6 was attached to the ankle (lateral malleolus), the infrared marker M7 was attached to the heel (lateral process of calcaneal tubercle), and, finally, the infrared marker M8 was attached to the toes (metatarsophalangeal joint) [57]. The Optotrak Certus was used for measuring the spatial positions of these markers: Mi = (x_i_, y_i_, z_i_), 1 ≤ i ≤ 8. 

For the evaluation of the proposed model, we used the publicly available dataset [62] that we established in our previous study [57]. This dataset contains linear accelerations acquired from four off-the-shelf smartphones attached to the upper arm, hand, pelvis, and thigh, as shown in Figure 2. The spatial positions of 12 infrared markers attached to the smartphones Mi = (x_i_, y_i_, z_i_), 9 ≤ i ≤ 20, were measured on the treadmill. The reference stride lengths were calculated from the spatial positions of the infrared marker M7. 

The selection of walking speeds for the treadmill experiments was based on our previous study, in which experimentation included measuring the free walking of healthy adults [48]. Hereby, persons selected slow, normal, and fast walking speeds to their preference and tried to maintain the selected walking speed constant during trials. Mean values of average self-selected walking speeds during these trials led to the selection of walking speeds tested on the treadmill. Ergo, the value of 3.3 km/h was selected for slow walking speed, the value of 4.6 km/h for normal walking speed, and, finally, the value of 5.9 km/h for fast walking speed. Each treadmill trial lasted for approximately 15 min. Every person consequently spent approximately 45 min walking on the treadmill in total, testing previously listed slow, normal, and fast walking speeds. One supplementary set of experiments was conducted on a 21.64-meter-long rectangular-shaped test polygon, where free walking was tested. Here, persons chose their preferred walking speed during the trials and were asked to maintain it as constant as possible. The average walking speed was found to be 4.4 ± 0.6 km/h during these trials [57]. Similar to the treadmill experiments, each trial lasted for approximately 15 min again.

Ten persons (four women and six men) selected from a group of healthy adults participated in these experiments. Table 1 shows their demographic information. The persons’ age ranged from 19 to 32 years. Their height varied from 1.60 to 1.88 m, and their leg length ranged from 0.90 to 1.16 m. 

### 2.2. Derivation of the Model

This study aimed to investigate the possibilities of extending the acceleration-based model we proposed in our previous work [57]. This model utilizes acceleration magnitude as the predominant input and achieved promising results on par with compared acceleration-based models, yet it was affected by walking speed and produced less accurate results in trials on the test polygon where persons self-selected their walking speed [57]. For this reason, CCA was utilized to examine the correlation of the input parameters of the model, with potential parameters extracted from spatial positions of the infrared markers that tracked the movement of anatomical landmarks during walking. CCA is a technique for searching for pairs of linear functions between two groups of variables such that the correlation between the linear functions within each pair is maximized [64]. The steps of the derivation process are presented next. 

The basis of this work presented the model that calculates step length $d_{est}$ as

(1) $$d_{est}=K\cdot a_{r}^{0.1}$$

where K represents a tunable constant and $a_{r}$ the range of acceleration magnitude within the step. As walking speed affected the performance of this model, the link between the values of the tunable constant under different experimental circumstances and the parameters extracted from spatial positions of infrared markers that tracked the movement of anatomical landmarks during walking were analyzed next by means of CCA.

Before utilizing CCA, relevant data were extracted from the measurements acquired by infrared markers attached to anatomical landmarks on the human body during walking. The motion of the infrared marker can be represented as a curve in space where its position is represented by three components corresponding to triaxial position coordinates that change with regard to time [65]. If the time difference between two consecutive samples is small enough, the velocity vector can be defined as the derivative of the position with regard to time. Similarly, the acceleration vector can be defined as the derivatives of the velocity with regard to time. Finite difference formulas were employed for the approximation of numerical derivatives. Hereby, the central difference formula was used for interior data points, whereas single-sided differences were used for endpoints. Obtained derivatives were filtered with a moving average filter over 10 data points [57].

Next, potential parameters were determined. Hereby, the aim was to select a number of relevant parameters that can be determined from inertial sensor measurements without too complex calculations, such as integration, to avoid potential errors being propagated in the process. Data were divided into two matrices, $X_{1}$ and $X_{2}$, for each trial and infrared marker. The matrix $X_{1}$ included tunable constants K per stride, calculated from Equation (1) for each infrared marker, except for M7. The matrix $X_{2}$ included the following data: stride frequencies, durations of strides, and mean, median, and range values of acceleration and vectors for all dimensions. Each type of the listed data for matrix $X_{2}$ corresponds to one column, whereas the rows in matrices $X_{1}$ and $X_{2}$ match strides. CCA was utilized on input matrices $X_{1}$ and $X_{2}$ to obtain the canonical coefficients $A_{1}$ and $A_{2}$ that correspond to coefficients of linear functions, respectively. 

Since only tunable constants were included in matrix $X_{1}$, the corresponding matrix of canonical coefficients $A_{1}$ was not of interest. The main focus was on the matrix of canonical coefficients $A_{2}$ instead. Here, coefficients were observed to determine the parameter that had the greatest impact. It was concluded that stride frequency had the greatest impact due to the canonical coefficients contributing to the linear combination the most. Hence, this was the first parameter in the extended model. Still, the remaining parameters had to be tackled. Therefore, an additional tunable constant was included in the model resulting in

(2) $$d_{est}=\left(K_{1}\cdot F+K_{2}\right)\cdot a_{r}^{0.1}$$

where $d_{est}$ represents estimated walked distance, F stride frequency, $a_{r}$ the range of acceleration magnitude, and $K_{1}$ and $K_{2}$ tunable constants.

In addition, it was observed that stride frequency and acceleration magnitude inputs are highly correlated to walking speed. Altogether, three walking speeds were tested during the experiments for the derivation of the model. Based on the average values of acceleration magnitude and stride frequency inputs, tunable constants $K_{1}$ and $K_{2}$ can be dynamically determined for each walking speed and person for every *N* strides. This approach is highly personalized as it takes into account each person separately, yet it can contribute towards estimating walked distance more accurately. It was, therefore, utilized in the evaluation of this study to lay the groundwork for the future work of determining the values of tunable constants by means of one of the gait authentication techniques that can present the potential to be generalized over similar gait patterns. 

### 2.3. Performance Evaluation

The performance of the proposed model was evaluated utilizing the dataset from the publicly available benchmark repository for the evaluation of step length estimation models [62]. This dataset includes inertial sensor measurements for long-term walking of four off-the-shelf smartphones obtained when 10 participants walked on the treadmill and test polygon for different walking speeds. Several models were selected, implemented, and evaluated utilizing the same dataset. The performance evaluation with reference to stride length estimation accuracy for treadmill experiments and estimated walked distance accuracy for test polygon experiments was considered for all models.

The models had to meet certain criteria in order to be considered for the selection to be implemented, evaluated, and compared to the proposed model. According to the first criterion, the predominant sensor inputs of the model have to include both acceleration and step frequency inputs. Another criterion was the fact of sufficient description for the implementation being present. As a consequence, several representative models were selected for the comparison, i.e., models proposed by Sharp and Yu [61], Shin and Park [60], Bylemans et al. [31], and Mikov et al. [59]. Table 2 shows the characteristics of models selected for the evaluation, namely the inputs, equation for step length estimation, and basis. 

These models were implemented and then evaluated using the publicly available dataset established in the scope of our previous work [57,62]. The experimental details were summarized in Section 2.1. All data were used for the evaluation of the models. The latter were, therefore, evaluated for both treadmill and free walking for four smartphone positions and different walking speeds. For treadmill walking, these models were evaluated for slow, normal, and fast walking speeds with four off-the-shelf smartphones attached to the upper arm, hand, pelvis, and thigh. For free walking, these models were evaluated utilizing the data collected on the rectangular-shaped test polygon, where participants self-selected their walking speed and maintained it as similar as possible. Again, four off-the-shelf smartphones were attached to the upper arm, hand, pelvis, and thigh. 

As a result, the evaluation was conducted in two stages. In the first stage, data collected during the treadmill experiments were used. The first five minutes of data collected in each trial were utilized for the tuning of the models, while the remaining 10 min were utilized for the evaluation. Tunable constants of models were determined by employing the least square estimation method for each trial and smartphone position. One hundred and twenty sets of tunable constants were calculated for each model. In the second stage of the evaluation, the data collected on the polygon were used. The selected models were tuned by using the data collected during the first five minutes of the treadmill experiment. However, for each smartphone position and each person, the data collected for different walking speeds were joined. Again, the least square estimation method was used. Altogether, 40 sets of tunable constants were calculated.

The performance of the models was calculated as follows. For the treadmill experiments, the performance of the models was calculated as the absolute difference between the estimated and measured stride lengths. Whereas for the test polygon experiments, the performance of the models e was calculated as:

(3) $$e=\frac{\left|d_{est}-d\right|}{d}\cdot 100\%$$

where $d_{est}$ represents the walked distance as estimated by the model and d the measured walked distance.

## 3. Results

Herein, we present the evaluation results with reference to MAEs, standard deviations (SDs), and coefficients of variation (CVs). First, we introduce the evaluation results of the treadmill experiments starting with the overall results and results for different smartphone positions and different walking speeds. Later, we present the evaluation results of the polygon experiments.

### 3.1. Treadmill Experiment

#### 3.1.1. Overall Results

Table 3 presents the overall MAEs, SDs, and CVs of stride length estimation. MAEs range from 5.64 to 10.92 cm, whereas SDs range from 4.33 to 13.56 cm. CVs vary from 0.76 to 1.24. The proposed model outperformed all the models selected for the comparison in terms of the accuracy of stride length estimation. It produced an MAE of 5.64 cm, an SD of 4.94 cm, and a CV of 0.88. The model proposed by Shin and Park [60] performed slightly less accurately than the proposed model, producing an overall MAE of 5.67 cm and an SD of 4.33 cm. In addition, its SD is the lowest among all the models selected for comparison. Consequently, the value of CV is also the lowest. The model proposed by Sharp and Yu [61] yielded results similar to the previous two models. Its MAE is 5.94 cm and SD 4.64 cm, whereas its value of CV equals 0.78. Models proposed by Bylemans et al. [31] and Mikov et al. [59] performed the worst by producing MAEs of 8.02 and 10.92 cm and corresponding SDs of 7.27 and 13.56 cm, respectively. Attention should also be drawn to the model proposed by Mikov et al. [59], as it produced the most dispersed stride length estimation errors.

Table 4 summarizes percentage shares of overestimated and underestimated stride length. The percentage shares vary from 36.18 to 47.79% for overestimation and from 52.21 to 63.82% for underestimation, indicating that the selected models mainly underestimated stride lengths. The proposed model overestimated approximately 40% of stride lengths and underestimated approximately 60% of stride lengths, demonstrating performance commensurate to the other models. 

Table 5 shows the MAEs, SDs, and CVs of stride length overestimation and underestimation. The MAEs and SDs of overestimated stride lengths range from 5.61 to 10.53 cm and from 4.84 to 12.60 cm, respectively. The corresponding CVs range from 0.86 to 1.20. Similarly, the MAEs of underestimated stride lengths range from 5.52 to 11.26 cm, and the corresponding SDs from 4.01 to 14.32 cm. Here, the CVs vary from 0.70 to 1.27. All models except for the proposed model and the model proposed by Bylemans et al. [31] generally produced more accurate results for overestimation. The proposed model outperformed all models in terms of the accuracy of underestimated stride lengths. Other models yielded performance similar to the overall evaluation summarized in Table 3: models proposed by Shin and Park [60], Sharp and Yu [61], Bylemans et al. [31], and Mikov et al. [59] produced MAEs of 5.70, 6.00, 7.14, and 11.26 cm, respectively. The model proposed by Shin and Park [60] produced an MAE of 5.61 cm and SD of 4.84 cm, outperforming all models when considering stride length overestimation. It also produced the lowest CV. The proposed model and the model proposed by Sharp and Yu [61] performed quite similarly in terms of the accuracy of stride length estimation. They produced MAEs of 5.77 and 5.82 cm, respectively. Again, models proposed by Bylemans et al. [31] and Mikov et al. [59] performed the worst when considering stride length overestimation. 

To sum up, the proposed model yielded MAEs of 5.52 cm and 5.77 cm in the scope of overall evaluation when considering stride length underestimation and overestimation, respectively. The obtained CVs are also in line with the values of CVs produced by other models, as the values of CVs of the proposed model are not greater or lower than the minimum and maximum values of CVs produced by other models. Similar conclusions can be drawn for the SDs and the percentage shares of overestimation and underestimation produced by the proposed model as well. For these reasons, we do not distinguish between overestimated and underestimated stride lengths henceforth and only present the overall MAEs, corresponding SDs, and CVs for different walking speeds and smartphone positions. 

#### 3.1.2. Smartphone at Upper Arm

**Figure 3:**
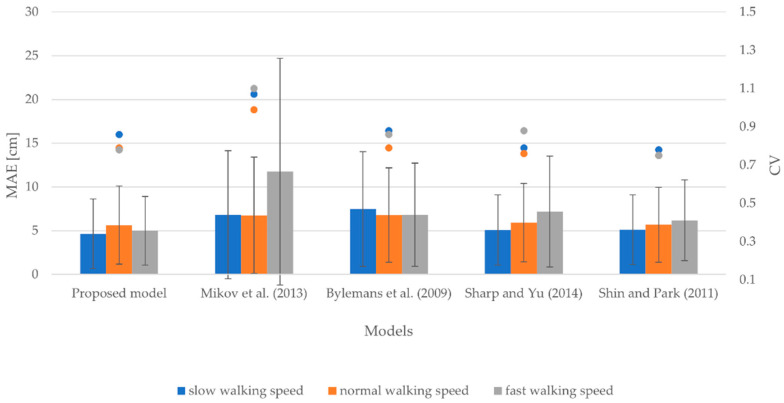
MAEs, SDs, and CVs for slow, normal, and fast walking speeds considering the smartphone attached to the upper arm.

Figure 3 includes the MAEs, SDs, and CVs for small, normal, and fast walking speeds considering the smartphone attached to the upper arm. The MAEs range from 4.65 to 7.47 cm for slow walking speed, from 5.64 to 6.78 cm for normal walking speed, and from 4.99 to 11.75 cm for fast walking speed. Similarly, the SDs vary between 3.97 and 7.31 cm for slow walking speed, 4.28 and 6.66 cm for normal walking speed, and 3.91 and 12.98 cm for fast walking speed. The CVs range from 0.86 to 1.07 for slow walking speed, from 0.75 to 0.99 for normal walking speed, and from 0.75 to 1.10 for fast walking speed. 

The results indicate that the proposed model outperformed other models in terms of the accuracy of stride length estimation for all walking speeds. It produced MAEs of 4.65, 5.64, and 4.99 cm for slow, normal, and fast walking speeds, respectively. Of note is that models yielded the most accurate results for normal walking speed on average. Notably, the model proposed by Shin and Park [60] demonstrated the lowest degree of variability among models on average. In contrast, the model proposed by Mikov et al. [59] achieved the highest degree of variability. 

#### 3.1.3. Smartphone at Hand

**Figure 4:**
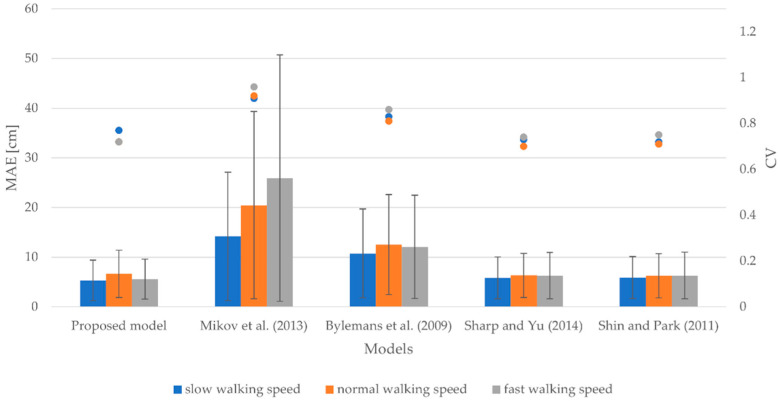
MAEs, SDs, and CVs for slow, normal, and fast walking speeds considering the smartphone attached to the hand.

Figure 4 shows the evaluation results for the smartphone attached to the hand. It demonstrates the MAEs, SDs, and CVs for slow, normal, and fast walking speeds. More specifically, MAEs vary between 5.32 and 14.18 cm and SDs vary between 4.09 and 12.95 cm for a slow walking speed. For normal and fast walking speeds, the MAEs range from 6.25 to 20.46 cm and from 5.58 to 25.92 cm, respectively, whereas the corresponding SDs vary from 4.42 to 18.85 cm for a normal walking speed and from 4.01 to 24.82 cm for a fast walking speed. The CVs range from 0.72 to 0.91 for a slow walking speed, from 0.70 to 0.92 for a normal walking speed, and from 0.72 to 0.96 for a fast walking speed.

The obtained results indicate that the proposed model yielded the most accurate results for slow and fast walking speeds in terms of accuracy of stride length estimation. Nevertheless, it was slightly outperformed by the models proposed by Sharp and Yu [61] and Shin and Park [60] for a normal walking speed. The MAE of the proposed model hereby is approximately 0.30 cm greater than the MAEs of the models proposed by Sharp and Yu [61] and Shin and Park [60]. The models proposed by Bylemans et al. [31] and Mikov et al. [59] performed the worst for all walking speeds in terms of accuracy of stride length estimation. All the models, except for the models proposed by Mikov et al. [59] and Bylemans et al. [31], exhibited a similar degree of variability by producing CVs in the range of 0.70 to 0.77 for all walking speeds.

#### 3.1.4. Smartphone at Pelvis

**Figure 5:**
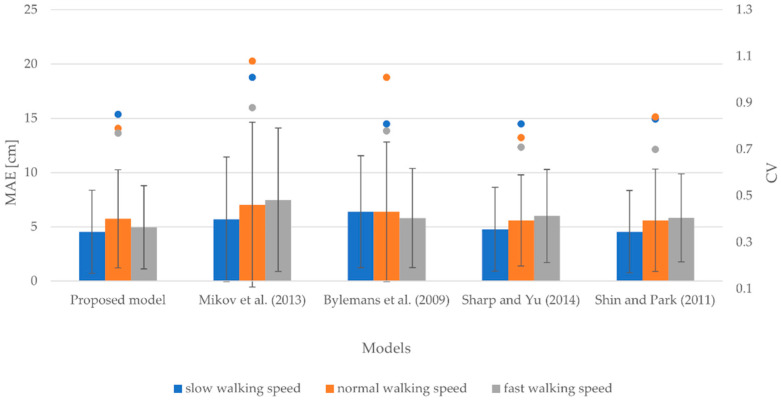
MAEs, SDs, and CVs for slow, normal, and fast walking speeds considering the smartphone attached to the pelvis.

Figure 5 demonstrates the evaluation results for the smartphone attached to the pelvis. It includes the MAEs, SDs, and CVs for slow, normal, and fast walking speeds. The MAEs range from 4.54 to 6.39 cm, from 5.59 to 7.05 cm, and from 4.95 to 7.49 cm for slow, normal, and fast walking speeds, respectively. Furthermore, the SDs are in the range of 3.79 to 5.74 cm, 4.20 to 7.59 cm, and 3.83 to 6.61 cm for slow, normal, and fast walking speeds, respectively. The values of CVs are between 0.81 and 1.01 for a slow walking speed, between 0.75 to 1.08 for a normal walking speed, and between 0.70 and 0.88 for a fast walking speed.

The results indicate that the proposed model outperformed all the other models for slow and fast walking speeds in terms of accuracy of stride length estimation, whereas the model proposed by Sharp and Yu [61] achieved the most accurate results of stride length estimation for a normal walking speed. Hereby, the proposed model produced an MAE of 5.74 cm, which is only 0.15 cm greater than the MAE produced by the model proposed by Sharp and Yu [61]. The models proposed by Sharp and Yu [61] and Shin and Park [60] yielded similar performances in terms of stride length estimation accuracy for different walking speeds. The model proposed by Bylemans et al. [31] estimated stride lengths least accurately for a slow walking speed, whereas the model proposed by Mikov et al. [59] estimated stride lengths least accurately for normal and fast walking speeds. Notably, models proposed by Bylemans et al. [31] and Mikov et al. [59] demonstrated the highest degree of variability among models on average by producing CVs between 0.78 and 1.08.

#### 3.1.5. Smartphone at Thigh

**Figure 6:**
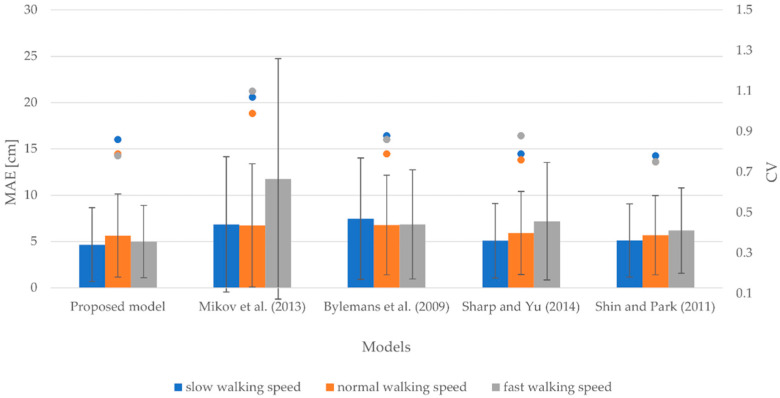
MAEs, SDs, and CVs for slow, normal, and fast walking speeds considering the smartphone attached to the thigh.

Figure 6 lays out the evaluation results for the smartphone attached to the thigh, assembling MAEs, SDs, and CVs of the models for slow, normal, and fast walking speeds. The MAEs vary between 4.65 and 7.47 cm for a slow walking speed, whereas the corresponding SDs range from 3.97 to 7.31 cm. The MAEs vary from 5.64 to 6.78 cm and the SDs from 4.28 to 6.66 cm for a normal walking speed. Furthermore, the MAEs range from 4.99 to 11.75 cm for a fast walking speed, and the corresponding SDs vary between 3.91 to 12.98 cm. The CVs range from 0.78 to 1.07, from 0.75 to 0.99, and from 0.75 to 1.10 for slow, normal, and fast walking speeds, respectively. 

The results indicate that our proposed model outperformed all the other models selected for comparison in terms of the accuracy of stride length estimation. The model proposed by Bylemans et al. [31] estimated stride lengths least accurately for slow and normal walking speeds. Similarly, the model proposed by Mikov et al. [59] estimated stride lengths least accurately for a fast walking speed. The models proposed by Shin and Park [60] and Sharp and Yu [61] yielded similar performances in terms of the accuracy of stride length estimation when considering slow and normal walking speeds. Again, the model proposed by Mikov et al. [59] exhibited the highest degree of variability among the models on average by producing CVs in the range of 0.99 to 1.07. Notably, the model proposed by Shin and Park [60] produced the least dispersed stride length estimation errors, whereas the CVs between 0.78 and 0.88 produced by the models proposed by Sharp and Yu [61] and Bylemans et al. [31] indicate that these models exhibit a similar degree of variability in terms of the accuracy of stride length estimation.

### 3.2. Polygon

#### 3.2.1. Overall Results

Table 6 shows the overall evaluation results of walked distances as estimated by the selected models in terms of MAEs, SDs, and CVs. The MAEs range from 4.55 to 13.73%, whereas the SDs range from 4.03 to 8.41%. The CVs vary between 0.53 and 1.02. The results indicate that the proposed model outperformed all the other models selected for the comparison in terms of the accuracy of the estimated walked distance on average. Similar to the proposed model, models proposed by Sharp and Yu [61] and Shin and Park [60] also produced MAEs within 10% of the walked distance. The previously listed three models also produced CVs with the greatest values indicating the largest degree of variability among models selected for the comparison. In contrast, models proposed by Bylemans et al. [31] and Mikov et al. [59] achieved MAEs greater than 10% and CVs with the lowest values.

#### 3.2.2. Results for Different Smartphone Positions

**Figure 7:**
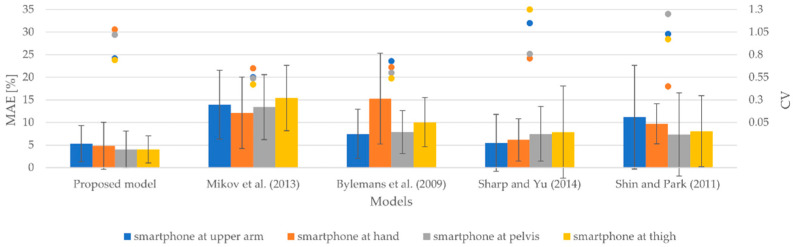
MAEs, SDs, and CVs of estimated walked distances for smartphones attached to the upper arm, hand, pelvis, and thigh.

Figure 7 lays out the results produced by the models for smartphones attached to the upper arm, hand, pelvis, and thigh. It includes MAEs that range from 5.32 to 13.94%, from 4.83 to 15.24%, from 4.01 to 13.41%, and from 4.04 to 15.42% for smartphones attached to the upper arm, hand, pelvis, and thigh, respectively. Moreover, the SDs vary from 4.04 to 11.47%, from 4.41 to 10.04%, from 4.09 to 9.19%, and from 3.00 to 10.18% for smartphones attached to the upper arm, hand, pelvis, and thigh, respectively. The CVs achieved by the models vary between 0.55 to 1.15, between 0.45 and 1.08, between 0.54 and 1.25, and between 0.47 to 1.30 for smartphones attached to the upper arm, hand, pelvis, and thigh, respectively.

The results indicate that the proposed model outperformed all the models selected for the comparison for all smartphone positions in terms of accuracy of estimated walked distance on average. Notably, the model proposed by Sharp and Yu [61] produced an MAE of 5.48% for the upper arm position, which is greater only by 0.16% than the MAE of the proposed model, yet the performance of this model deteriorated for other smartphone positions when considering the accuracy of the estimation of walked distance. The model proposed by Bylemans et al. [31] estimated the walked distance least accurately for the smartphone attached to the hand, whereas for other positions, the model proposed by Mikov et al. [59] yielded the least accurate results in terms of estimated walked distance. Notably, the models proposed by Bylemans et al. [31] and Mikov et al. [59] produced CVs with the lowest values for all smartphone positions except when the smartphone was attached to the hand. This indicates the lowest degree of variability among models. For the smartphone attached to the hand, the model proposed by Shin and Park [60] produced the lowest CV. In addition, this model’s CVs are greater more than twice that the CV for the hand position. The model proposed by Sharp and Yu [61] exhibited the greatest degree of variability when smartphones were attached to the upper arm and thigh. The CVs achieved by the proposed model are in line with the CVs produced by the other models, as the values of CVs of the proposed model are not greater or lower than the minimum and maximum values of CVs produced by other models.

## 4. Discussion

We discuss the evaluation results of the selected models herein. First, a comparison of models is accomplished based on their characteristics. Later, the evaluation results for the treadmill and polygon experiments are discussed, considering different walking speeds and smartphone positions. Finally, future research directions conclude this section. 

### 4.1. Comparison of the Models

The outcome of this study is an enhanced step length estimation model that employs acceleration magnitude and step frequency inputs. Smartphone orientation does subsequently not affect step length estimation, unlike the models proposed by Mikov et al. [59], Bylemans and al. [31], and Sharp and Yu [61]. These models utilize vertical acceleration and step frequency inputs. In addition, the model proposed by Sharp and Yu [61] also includes the user’s height. The model proposed by Shin and Park [60] is unaffected by smartphone orientation as well, as it utilizes step frequency and acceleration magnitude inputs very similarly to the proposed model. The proposed model utilizes the difference between maximum and minimum acceleration magnitude values within a step, whereas the model proposed by Shin and Park [60] employs acceleration variance. All the models are affected by smartphone position as they employ acceleration as one of the inputs. The spatiotemporal and kinematic parameters vary for the different walking speeds and body segments [66].

A number of models selected for the comparison are extensions of previously proposed models. The model proposed by Weinberg [55] laid the groundwork for the model proposed by Mikov et al. [59], the model proposed by Kim et al. [56] laid the groundwork for the model proposed by Bylemans et al. [31], and the model proposed by Vezočnik et al. [57] laid the groundwork for the proposed model. The models proposed by Shin and Park [60] and Sharp and Yu [61] are the results of the analysis and observations conducted by the authors.

The models also employ a different number of tunable constants. For instance, the models proposed by Mikov et al. [59] and Bylemans et al. [31] include one tunable constant. The model proposed by Shin and Park [60] includes three tunable constants, and the model proposed by Sharp and Yu [61] employs four tunable constants. The proposed model includes two tunable constants. 

While all the models can be used in pedestrian navigation using inertial sensors, only the proposed model and the model proposed by Shin and Park [60] are unaffected by smartphone orientation due to their predominant inputs being acceleration magnitude and step frequency. The proposed model has one tunable constant fewer than the model proposed by Shin and Park [60] and can, therefore, present an option for step length estimation. 

### 4.2. Evaluation of Walking on the Treadmill 

The overall results obtained from the treadmill experiment data indicate that the proposed model outperformed all the models selected for the comparison. Table 2 demonstrates that our model estimated stride lengths more precisely than the other models by producing an overall MAE of 5.64 cm. The model proposed by Shin and Park [60] exhibited performance similar to the proposed model. However, its overall MAE was greater than the MAE of the proposed model by 0.03 cm. The similar performance might be the consequence of similar inputs as both models utilize acceleration magnitude and step frequency. Just like the previous two models, the model proposed by Sharp and Yu [61] produced an overall MAE lower than 6.00 cm, yet it was slightly worse. The models proposed by Bylemans et al. [31] and Mikov et al. [59] yielded less accurate overall results. Notably, the model proposed by Mikov et al. [59] produced the largest MAE and SD. All models mainly underestimated stride lengths and did not exhibit any significant difference for stride length overestimation and underestimation. 

The results achieved from the proposed model indicate that it outperformed all the models for slow and fast walking speeds and all smartphone positions in terms of stride length estimation accuracy. Similarly, it also outperformed the other models for a normal walking speed when considering smartphones attached to the upper arm and thigh, whereas the model proposed by Shin and Park [60] estimated stride lengths slightly more accurately than the proposed model for smartphones attached to the hand and pelvis for normal walking speed. Overall, the proposed model exhibited steady performance for different walking speeds and smartphone positions in terms of accuracy of stride length estimation.

The walking speed affected the performance of the models. The models proposed by Shin and Park [60] and Sharp and Yu [61] achieved less accurate and varying results as the walking speed increased. The model proposed by Mikov et al. [59] performed similarly to these two models in terms of stride length estimation accuracy when smartphones were attached to the hand and pelvis, yet it produced slightly less accurate and less disperse results. When smartphones were attached to the upper arm and thigh, this model produced very similar stride length estimation results for slow and normal walking speeds. However, the performance of this model significantly deteriorated for a fast walking speed. The model proposed by Bylemans et al. [31] estimated stride lengths least accurately among models for a slow walking speed for all smartphone positions except for the smartphone attached to the hand. Our proposed model was also affected by walking speed since it yielded less precise results for a normal walking speed in general. Even though the results of the proposed model do not differ from the results produced by the models proposed by Shin and Park [60] and Sharp and Yu [61], the obtained CVs of the proposed model indicate that the results are indeed more dispersed. However, the model proposed by Shin and Park includes one tunable constant more than the proposed model, and the model proposed by Sharp and Yu [61] include two tunable constants more than the proposed model, as well as the user’s height. This might have contributed to the advantage in terms of the extent of variability of estimation accuracy. 

### 4.3. Evaluation of Walking in the Polygon

The overall results indicate that the proposed model outperformed all the models selected for the comparison. It produced an overall MAE of 4.55%, which is more than two percent lower than the MAE of the second-best model proposed by Sharp and Yu [61]. When considering the average walked distance of 1128.81 m per trial in the polygon, two percent correspond roughly to 22.58 m. The model proposed by Shin and Park [60] produced an MAE almost twice as large as the proposed model. The models proposed by Bylemans et al. [31] and Mikov et al. [59] produced MAEs greater than 10.00%. Again, the latter model produced the least accurate results of estimated walked distance.

The proposed model demonstrated consistent performance for different smartphone positions. The walking speed also had the lowest impact on our proposed model. In general, the models produced results that were distributed around the overall MAE when considering different smartphone positions. Notably, the model proposed by Bylemans et al. [31] achieved significantly less accurate results of estimated walked distances when the smartphone was attached to the hand. However, the results of this model and the model proposed by Mikov et al. [59] were less dispersed than those produced by other models. Notably, the models proposed by Shin and Park [60] and Sharp and Yu [61] exhibited the largest extent of variability among the models. 

### 4.4. Future Research Directions

The main findings of our study are as follows. The obtained results indicate that our proposed model outperformed the models selected for the comparison. Moreover, smartphone orientation has no effect on the proposed model due to the predominant inputs being acceleration magnitude and step frequency. As a consequence, no particular deliberation is needed for the smartphone placement on the person’s body. The proposed model can, therefore, represent a reasonable choice for estimating step length in PDR-based approaches. 

One limitation of this study is related to the absence of scenarios typical for smartphone users due to tracking the smartphone positions with the optical measurement system. Another limitation corresponds to the number of persons included in the experiments. The adaptive design of the proposed model is highly accurate, yet it is also highly personalized due to requiring it to be tailored for each individual. We have dedicated a certain amount of time to determine the optimal values of tunable constants, as they had to be calculated for different walking speeds. As a consequence, we applied the optimization method several times. Moreover, we also stored average acceleration magnitude values and stride frequency inputs for each walking speed, as they were used as the basis for the selection of tunable constants in the step length estimation procedure. Nevertheless, several studies have already proved that inertial sensors can be utilized for gait recognition [67]. This is in line with the design of the model. For this reason, one future research direction could be investigating the link between tunable constants and gait signals, and considering different smartphone positions to develop an algorithm that could be used for step length estimation without prior tuning. Conducting additional experiments and including more persons could represent one step towards the establishment of a reference database that could be used to automatically tune the proposed model without prior tuning by considering only gait signals. 

## 5. Conclusions

In this paper, we presented an enhanced step length estimation model derived by means of CCA. Spatial positions of anatomical landmarks on the human body during walking, tracked by an optical measurement system, were utilized in the derivation process. The proposed model utilizes acceleration magnitude as well as step frequency inputs. Consequently, smartphone orientation does not affect the proposed model. When considering the average values of inputs, tunable constants can be dynamically determined for every predefined number of strides, thus enabling the model to adapt to changes in walking speed per person.

We evaluated the proposed model by using a publicly available dataset [62] from our open repository for evaluation. This dataset includes measurements for 10 persons collected on four off-the-shelf smartphones for long-term walking and considering different walking speeds on the treadmill and the rectangular-shaped polygon. The proposed model achieved an overall MAE of 5.64 cm on the treadmill and an overall mean walked distance error of 4.55% on the test polygon, outperforming all the models selected for comparison. It can, therefore, present a reasonable choice for estimating step length in PDR-based approaches.

## Figures and Tables

**Table 1:** Demographic information of persons.

Age Groups [in Years]	Gender		Height	Leg Length
	Male	Female		
19–25	3	0	1.81–1.88 m (mean value of 1.86 ± 0.04 m)	1.09–1.16 m (mean value of 1.12 ± 0.04 m)
26–32	3	4	1.60–1.83 m (mean value of 1.73 ± 0.09 m)	0.90–1.09 m (mean value of 1.02 ± 0.06 m)

**Table 2:** Characteristics of models selected for the evaluation.

Models	Input	Equation	Basis
Mikov et al. [59]	tunable constant K, step frequency F, maximum vertical acceleration value within a step $a_{max}$, minimum vertical acceleration value within a step $a_{min}$	$\frac{K}{F}\;\cdot \;\sqrt[{4}]{a_{max}-a_{min}}$	the model proposed by Weinberg [55]
Bylemans et al. [31]	tunable constant K, step frequency F, maximum vertical acceleration value within a step $a_{max}$, minimum vertical acceleration value within a step $a_{min}$, mean absolute vertical acceleration value within a step $a_{mean}$	$0.1\cdot \sqrt[{2.7}]{a_{mean}\cdot \sqrt{K\sqrt{\frac{F}{a_{max}-a_{min}}}}}\;$	the model proposed by Kim et al. [56]
Shin and Park [60]	tunable constants $K_{1}$, $K_{2}$, and $K_{3}$, acceleration magnitude variance within a step $a_{v}$, step frequency F	$K_{1}\cdot F+K_{2}\cdot a_{v}+K_{3}$	influence of step frequency and acceleration magnitude variance on step length
Sharp and Yu [61]	tunable constants $K_{1}$, $K_{2}$, $K_{3}$, and $K_{4}$, user’s height h, step frequency F, maximum vertical acceleration value within a step $a_{max}$, mininumum vertical acceleration value within a step $a_{min}$	$K_{1}\cdot h^{K_{2}}\cdot {\left(a_{max}-a_{min}\right)}^{K_{3}}\cdot F^{K_{4}}$	relation between step length and user’s height, step frequency and the difference between the maximum and minimum vertical acceleration values within the step

**Table 3:** Overall MAEs, SDs, and CVs of stride length estimation.

Models	MAE [cm]	SD [cm]	CV
Proposed model	5.64	4.94	0.88
Mikov et al. [59]	10.92	13.56	1.24
Bylemans et al. [31]	8.02	7.27	0.91
Sharp and Yu [61]	5.94	4.64	0.78
Shin and Park [60]	5.67	4.33	0.76

**Table 4:** Percentage shares of overestimated and underestimated strides lengths.

Models	Overestimation [%]	Underestimation [%]
Proposed model	39.99	60.01
Mikov et al. [59]	46.20	53.79
Bylemans et al. [31]	47.79	52.21
Sharp and Yu [61]	36.18	63.82
Shin and Park [60]	36.38	63.62

**Table 5:** MAEs, SDs, and CVs of stride length overestimation and underestimation.

Models	Overestimation			Underestimation		
	MAE [cm]	SD [cm]	CV	MAE [cm]	SD [cm]	CV
Proposed model	5.77	5.26	0.91	5.52	4.09	0.74
Mikov et al. [59]	10.53	12.60	1.20	11.26	14.32	1.27
Bylemans et al. [31]	8.98	8.38	0.93	7.14	5.95	0.83
Sharp and Yu [61]	5.82	5.09	0.87	6.00	4.35	0.73
Shin and Park [60]	5.61	4.84	0.86	5.70	4.01	0.70

**Table 6:** Overall MAEs, SDs, and CVs of estimated walked distances achieved by the models.

Models	MAE [%]	SD [%]	CV
Proposed model	4.55	4.03	0.89
Mikov et al. [59]	13.73	7.30	0.53
Bylemans et al. [31]	10.17	7.21	0.71
Sharp and Yu [61]	6.74	6.89	1.02
Shin and Park [60]	9.08	8.41	0.93

## Data Availability

Evaluation data are publicly available at the open repository for evaluation at https://github.com/repositoryadmin/SLERepository (accessed on 29 November 2022).
